# Emergent rogue wave structures and statistics in spontaneous modulation instability

**DOI:** 10.1038/srep10380

**Published:** 2015-05-20

**Authors:** Shanti Toenger, Thomas Godin, Cyril Billet, Frédéric Dias, Miro Erkintalo, Goëry Genty, John M. Dudley

**Affiliations:** 1 Institut FEMTO-ST, UMR 6174 CNRS-Université de Franche-Comté, Besançon, France; 2 School of Mathematical Sciences, University College Dublin, Belfield, Dublin 4, Ireland; 3 Department of Physics, University of Auckland, Auckland, New Zealand; 4 Department of Physics, Tampere University of Technology, Tampere, Finland

## Abstract

The nonlinear Schrödinger equation (NLSE) is a seminal equation of nonlinear physics describing wave packet evolution in weakly-nonlinear dispersive media. The NLSE is especially important in understanding how high amplitude “rogue waves” emerge from noise through the process of modulation instability (MI) whereby a perturbation on an initial plane wave can evolve into strongly-localised “breather” or “soliton on finite background (SFB)” structures. Although there has been much study of such structures excited under controlled conditions, there remains the open question of how closely the analytic solutions of the NLSE actually model localised structures emerging in noise-seeded MI. We address this question here using numerical simulations to compare the properties of a large ensemble of emergent peaks in noise-seeded MI with the known analytic solutions of the NLSE. Our results show that both elementary breather and higher-order SFB structures are observed in chaotic MI, with the characteristics of the noise-induced peaks clustering closely around analytic NLSE predictions. A significant conclusion of our work is to suggest that the widely-held view that the Peregrine soliton forms a rogue wave prototype must be revisited. Rather, we confirm earlier suggestions that NLSE rogue waves are most appropriately identified as collisions between elementary SFB solutions.

The terminology of “rogue wave” in physics describes events with high amplitude that emerge randomly in the dynamical behaviour of a particular system with low probability. This label was initially applied to describe the unexpected appearance of large and destructive waves on the ocean^1^,^2^ but has now been generalized to describe large amplitude rare events in many other systems^3^,^4^. Particular interest in rogue waves emerging during propagation in systems described by the nonlinear Schrödinger equation (NLSE) or its extensions has led to studies of rogue wave behaviour for deep water wave groups, pulse propagation in optical fibres, plasmas and cold atoms^5^,^6^,^7^.

The NLSE has particular significance in the context of rogue wave behaviour because it exhibits the Benjamin-Feir or modulation instability (MI), where a weak modulation on a plane wave will undergo exponential growth with propagation^8^,^9^. After this initial exponential growth, the subsequent dynamics sees periodic growth and decay in a form of Fermi-Pasta-Ulam (FPU) recurrence^10^. Because rapid growth and decay of a weakly modulated pulse envelope would also increase the amplitude and steepness of an underlying carrier wave, MI has long been considered a primary candidate for a rogue wave generating mechanism^11^,^12^,^13^.

Although the initial mathematical studies of MI were performed using linear stability analysis^9^, MI and FPU dynamics in the NLSE can also be described using various types of “breather” or soliton on finite background (SFB) solutions to the NLSE^14^,^15^,^16^,^17^. The possibility to describe these dynamics analytically has motivated much research to obtain possible insights into the particular initial conditions that may favour rogue wave emergence. For this reason, experiments in both optical fibre systems and hydrodynamic wave tanks have been carried out to generate particular SFB solutions using controlled initial conditions^4^,^7^.

A natural question that arises at this point, however, is how these SFB solutions are manifested in a genuinely chaotic MI field; or more precisely, how they may be related to coherent localised structures that appear randomly when MI is excited from noise. This question is clearly of fundamental interest, but it is also of practical significance, as noise-induced MI is the initial phase of continuous wave supercontinuum generation in optics^18^. A better understanding of the spatio-temporal dynamics of MI may well have impact on the design of practical supercontinuum sources, particularly through the use of external seeding to enhance MI and supercontinuum bandwidth^19^,^20^.

Although important studies of this question have been previously carried out focusing on the particular analytic solutions that fit the highest-intensity events observed in a chaotic MI field^21^,^22^,^23^, the objective of this paper is to study this question in more depth through analyzing properties of the emergent peaks in chaotic MI over their full intensity range. Our main results are as follows. Firstly, we show that one can identify distinct types of localised structure in a chaotic MI field that are well-described by several different classes of analytic SFB solution - from elementary breathers at lower intensities to higher-order SFB solutions arising from the collisions between the elementary solutions at the highest intensities. By analyzing the properties of a large number of peaks in the chaotic field, we are also able to confirm that the numerical results for the various classes of SFB observed scatter statistically about the expected analytic solutions. Secondly, we analyze in detail the statistical distribution properties of the peak intensities of the MI field, and we find a highly-skewed distribution with a very large fraction of the emergent peaks (~94%) having intensities below the intensity of the Peregrine soliton. This suggests that chaotic MI is dominated by elementary SFB solutions of the NLSE. Our results also indicate that the Peregrine soliton is not in fact statistically rare enough to be considered as a rogue wave prototype. Rather, our analysis confirms the earlier suggestions in Ref. 22 that NLSE rogue waves are most appropriately identified as collisions between elementary SFB solutions.

## Modulation Instability and Solitons on Finite Background

**Figure 1:**
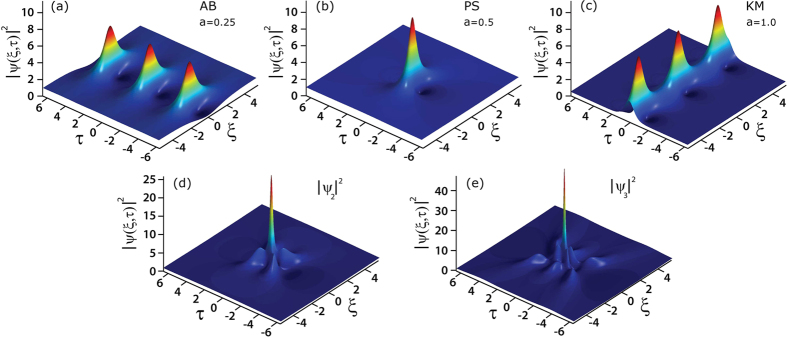
SFB solutions derived from Eq. (2) for different values of the parameter *a* as indicated: (**a**) Akhmediev breather (AB). (**b**) Peregrine soliton (PS). (**c**) Kuznetsov-Ma (KM) soliton. (**d**) and (**e**) second- and third-order solutions for the case of maximum intensity.

For completeness, we first present a brief review of the analytic SFB solutions, starting from the dimensionless focusing NLSE:
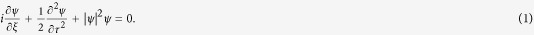
Here, the amplitude envelope *ψ* (*ξ*,*τ*) is a function of propagation distance *ξ* and co-moving time *τ*. In the context of our discussion below, it is convenient to consider the SFB solution to Eq. (1) written in the following form^14^,^25^:
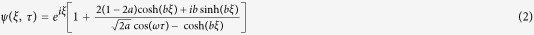


The properties of this solution are determined by one single parameter *a* (*a* ≠ 1/2), through *b *= [8*a* (1−2*a*)]^1/2^ and *ω* = 2(1−2*a*)^1/2^. Figure 1 shows how, as the parameter *a* varies, different behaviour in the solution is seen with localisation in space, time, or both. Over the range 0 < *a* < 1/2, Eq. (2) describes the Akhmediev breather (AB), a temporally-periodic pulse train that undergoes a single growth-decay cycle along *ξ*^21^. The temporal period of the AB pulse train is *Δτ* = *π*/(1−2*a*)^1/2^. It is the growth phase of the AB solution that also describes the initial exponential growth of a weak perturbation in the framework of MI. In this case, the real parameter *ω* gives the frequency of modulation on the perturbed plane wave, and the real parameter *b* governs the instability growth rate which is maximum for *a* = 1/4. At this maximum of MI gain, the temporal periodicity is *Δτ* = 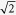
*π* and the maximum value of the AB intensity is |*ψ* (0,0)|^2^ = (1 + 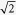)^2^ ~ 5.8. In this context, we note that for consistency with the many recent studies of NLSE and MI rogue wave solutions in optics, we plot and analyze our results in terms of intensity and not amplitude^4^.

The maximum intensity of the AB solutions increases with *a.* In the limit *a *→* *1/2, the solution as written in Eq. (2) is replaced by the rational Peregrine soliton (PS) solution: *ψ* (*ξ*,*τ*) = [1–4(1 + 2i*ξ*)/(1 + 4*τ*^2^ + 4*ξ*^2^)] *e*^i*ξ*^ 15. The PS solution possesses the maximum intensity amongst all the family of AB solutions with 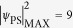. Equation (2) remains a solution of the NLSE even for *a* > 1/2, although the physical nature of the solution changes as circular trigonometric functions become hyperbolic and vice versa. In this case, we obtain the Kuznetsov-Ma (KM) soliton, which shows localisation in time *τ* but periodicity along *ξ*^16^,^26^.

The AB, PS and KM solutions can be referred to as “elementary” SFB solutions of the NLSE, but it is also possible to obtain more complex higher-order solutions using mathematical techniques such as the Darboux transformation^25^. These solutions possess even stronger localisation characteristics (along both time and space dimensions) and higher intensities than the PS limit. From a physical viewpoint, the higher-order solutions can be interpreted as a nonlinear superposition or collision of elementary breather solutions, with their exact functional form depending on the effective value of the *a*-parameter for each elementary constituent breather, as well as their relative phase difference and spatio-temporal position. The maximum possible intensity of the higher-order solutions occurs when one has nonlinear superposition of Peregrine soliton components with zero phase difference. In this case the solutions can be written in rational form, and the maximum intensity is 
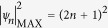
 where *n* is the (integer) solution order or, equivalently, the number of elementary constituent solitons^23^. (Note that when *n* = 1 we recover 
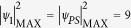
). Figure 1(d,e) show examples of such maximal-intensity higher order solutions for *n* = 2 and *n* = 3 respectively with 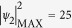 and 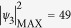.

## Noise-driven MI simulations—temporal and spectral characteristics

**Figure 2:**
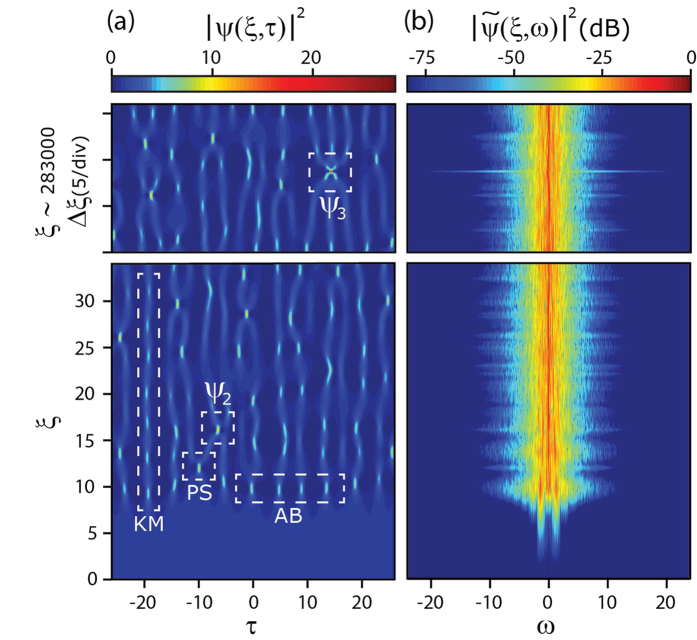
(**a**) Density map showing the long term temporal evolution of a chaotic field triggered by one photon per mode noise superimposed on a plane wave background. (**b**) Density map of the corresponding frequency evolution. Bottom subfigures plot evolution over *ξ* = 0 to *ξ* = 34; top subfigures plot evolution over a range around *ξ* ~ 283000.

In order to investigate whether these SFB structures also appear in a space-time MI field excited from noise, we performed extensive simulations of MI in the NLSE with a noise-perturbed plane wave initial condition (see Methods). Results showing the temporal (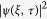) and spectral (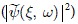) evolution of the chaotic MI field are shown in Fig. 2(a,b) respectively.

We first discuss the time-domain evolution map in Fig. 2(a). Here we see a series of temporal peaks developing as a result of the instability breaking up the input noisy plane wave. The peaks take the form of a quasi-periodic temporal pulse train when they first appear around *ξ* ~ 10, but further evolution is more chaotic and we observe various forms of interaction (merging, splitting) between individual cycles of the propagating breathers. Significantly, although these peaks emerge from noise, their temporal and spatial characteristics reveal clear signatures of the ideal analytic SFB solutions described above. For example, at the initial point of emergence around *ξ* ~ 10, the average temporal periodicity from the simulations is *Δτ* = 4.44, close to the calculated AB periodicity at maximum MI gain (at *a* = 1/4) given by *Δτ *~ 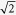
*π*.

**Figure 3:**
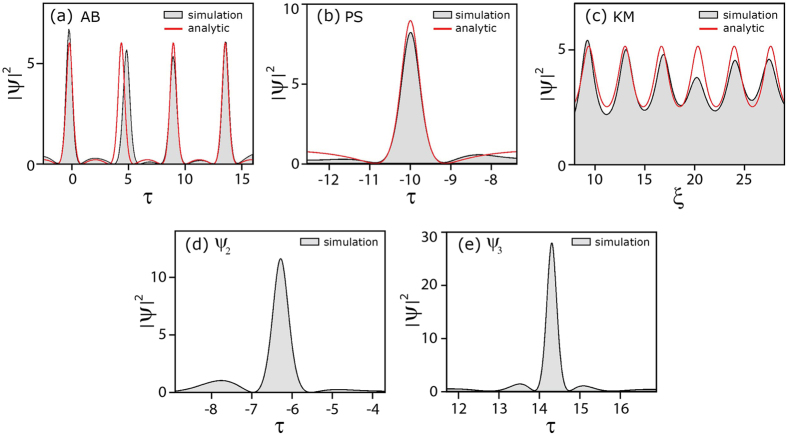
The gray shaded plots in (**a**,**b**,**c**) show the intensity profiles extracted from the regions of the chaotic MI field indicated in Fig. 2 for an AB, PS, KM respectively, compared with analytical NLSE solutions (red solid line). In (**d**) and (**e**) we show the intensity profiles extracted from the regions of the chaotic MI field indicated in Fig. 2 for a second-order superposition *ψ*_2_ and third-order superposition *ψ*_3_.

In addition, we can identify in the evolution map many other regions where the local properties of the MI field are well-fitted by the analytic SFB solutions given above. In particular, Fig. 3(a–c) compares the intensity profiles from the regions labelled AB, PS, and KM in Fig. 2(a) with the corresponding analytic SFB solutions. In Fig. 3(a,b) for the AB and PS regions, extracting the temporal intensity of each peak at the point of maximum localisation (gray shaded) yields an intensity profile agreeing very well with the corresponding calculated AB and PS solution (red line). In Fig. 3(c) for the KM region, plotting the evolution of the maximum intensity along *ξ* from simulation (gray shaded) agrees well with the analytic KM soliton results (red line). Of course, exact agreement is not to be expected given the random initial conditions, but these results very clearly show how the analytic solutions map very closely to the noise-generated structures.

As described in the Methods section, our simulations yielded a large ensemble of ~3 × 10^6^ distinct intensity peaks. This large data set has allowed us to identify higher order NLSE solutions with intensities greater than the 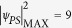 limit, and we label in Fig. 2(a) two such peaks. A peak arising from the superposition of two breathers is labelled 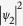 and a peak arising from the superposition of three breathers is labelled 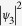. Figure 3(d,e) plot the corresponding temporal profiles at maximum localisation, showing respectively peak intensities of 11.5 for 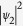
and 28 for 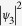, consistent with the identification as second-order and third- order solutions respectively. (Note that in this case because the constituent breather properties and their relative phase difference are unknown, we do not perform a comparison with any theoretical fit.)

**Figure 4:**
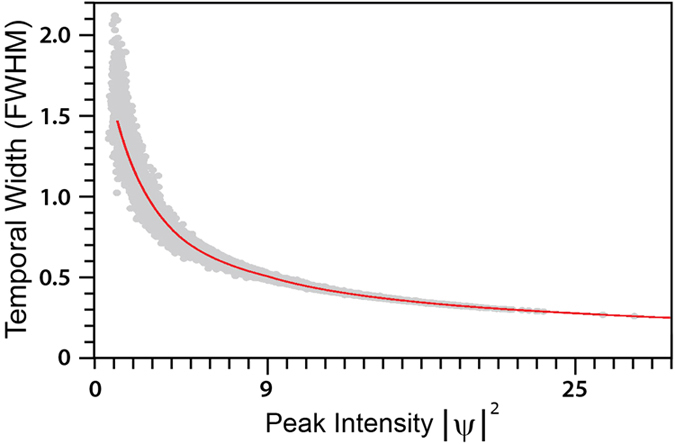
Scatter plot of temporal width (FWHM) against peak intensity for the 2853669 intensity peaks in the chaotic MI field from simulations (gray points) compared with theoretical predictions for the properties of SFBs (red solid line).

These results already strongly suggest a close correspondence between the properties of the analytic SFB solutions and the properties of the localised structures seen in noise-induced MI. Even more convincing evidence for this can be obtained from an analysis of *all* the peaks in the data set, comparing the temporal widths (FWHM) and peak intensities with those expected for SFB solutions including elementary and higher-order solutions. These results are shown in Fig. 4. Here, for each of the ~3 × 10^6^ peaks in the chaotic MI field, the scatter plot (gray points) shows the temporal FWHM plotted against the corresponding peak intensity. It is clear that the points span a continuous range of intensities up to a maximum intensity of ~28 in the region of third-order solutions.

We can compare this scatter plot with theoretical predictions for the properties of SFBs using the well-known analytic properties of AB solutions (for 0 < *a* < 1/2 corresponding to intensities <9), and by using the Darboux transformation to numerically construct higher-order solutions from superpositions of arbitrary elementary AB solutions (for intensities >9)^25^. For AB and higher-order SFB solutions calculated in this way, the solid line in Fig. 4 plots their computed temporal FWHM against maximum intensity; it is clear that the scattered points extracted from the chaotic MI field cluster closely around this calculated curve, providing additional confirmation that the analytic SFBs of the NLSE are indeed the appropriate framework to describe the emergent peaks in noise-induced MI. Note that the scatter about the calculated curve is to be expected since the calculated higher-order solutions assume only collisions with zero relative phase difference; the observed scatter arises from out-of-phase collisions.

**Figure 5:**
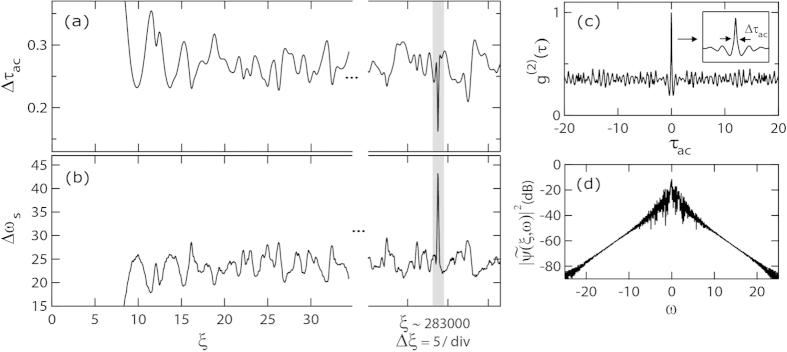
As a function of the propagation distance *ξ*, Figs 5(**a**) and (**b**) plot the evolution of the width of the autocorrelation coherence peak and the −80 dB spectral width for the evolving MI field in Fig. 2. These results illustrate how spectral expansion is associated with the appearance of shorter temporal structures in the random AB pulse train. Figs 5(**c**) and (**d**) show the autocorrelation and spectrum for the highest intensity peak associated with the collision between three breathers. The detail in (**c**) shows how the FWHM of the central autocorrelation coherence peak is determined.

Figure 4 also shows clearly how higher intensity peaks are associated with shorter temporal durations. This in fact suggests that the presence of higher-order solutions may also appear in the spectral domain through increased bandwidth. Although some suggestion of this can be seen from inspection of the temporal and spectral evolution in Fig. 2, we examine this quantitatively in Fig. 5 by plotting suitable measures of temporal and spectral width as a function of propagation distance. To characterize the temporal width of the evolving field, we calculate the intensity autocorrelation function: 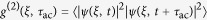 and compute the width *Δτ*_ac_ of the central coherence peak. This gives a measure of the average width of the localised temporal peaks in the noisy pulse train^27^. To determine a suitable measure of spectral width, we use the bandwidth *Δω*_s_ calculated at the −80 dB level in the wings of the spectrum (see Methods).

Figure 5(a,b) respectively plot the evolution of the width *Δτ*_ac_ of the central coherence peak and the bandwidth *Δω*_s_ as a function of distance *ξ*. Although both the temporal and spectral characteristics fluctuate significantly with propagation, we nonetheless can clearly see by inspection how larger spectral widths are associated with shorter autocorrelation coherence peaks and vice-versa. We highlight in particular (using shading) the point at which the structure with shortest temporal width and largest bandwidth emerges corresponding to the collision of 3 elementary breathers. At this distance, we also explicitly plot in Fig. 5(c,d) the autocorrelation and spectrum, with the inset in the autocorrelation function showing detail of the coherence peak (see Methods).

## Analysis of the Intensity Histogram

**Figure 6:**
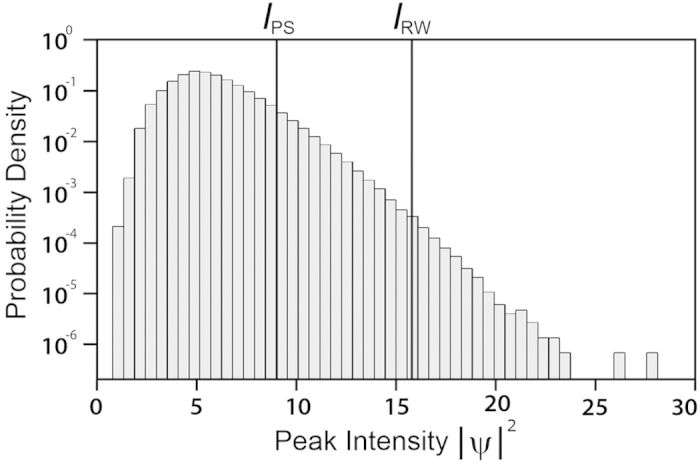
Peak intensity statistics obtained using specific peak detection over a full two-dimensional spatio-temporal computational window. The lines indicate the Peregrine soliton threshold *I*_PS_ 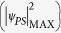 and the rogue intensity threshold *I*_RW_ (see text).

We gain further insight into the physical interpretation of the elementary and higher-order solutions by plotting a histogram of the intensities of the localised peaks in our ensemble. The result is shown in Fig. 6. The histogram shows a maximum at |*ψ* |^2^ ~ 5 after which the tails of the distribution decay exponentially (linearly on the semi-logarithmic scale used in the figure). It is particularly interesting to compute the integrated probability of observing peaks with intensity relative to that of the PS, the boundary between elementary and higher-order SFB solutions. This analysis yields that 93.8% of peaks have |*ψ* |^2^ < 9 corresponding to elementary SFBs, and 6.2% of peaks have higher intensities corresponding to the higher-order solutions.

This is a very significant result, since it is often suggested that the localisation properties of the Peregrine soliton are such that it is the “prototype” of rogue waves. Yet our results show in fact that Peregrine solitons do not appear in the extreme tails of the histogram with the low probabilities commonly associated with rogue waves. To make this point clearer, we adapt the criterion commonly used in hydrodynamics to define rogue waves by introducing a *rogue wave intensity* threshold *I*_RW_ defined as twice the “significant intensity,” the mean of the highest third of peaks. In this way, any localised structure with peak intensity |*ψ* |^2^ ≥ *I*_RW_ is considered as a candidate for a “rogue” event. For our simulation results, the rogue wave intensity *I*_RW_ ~ 15.8 is indicated by the vertical line in the histogram plot of Fig. 6, and based on this criterion, only 0.04% of the peaks in the ensemble have intensities exceeding this value. Such peaks can clearly be described as rare rogue wave events. Moreover, using this criterion, we can also make the more general statement that it is only the structures arising from collisions of elementary breathers that satisfy the definition of rogue wave events.

## Discussion and Conclusions

There are several conclusions to be drawn from the results in this paper. Firstly, our results extend previous studies in this area by studying the properties of coherent localised structures in a chaotic MI field over a large range of intensities – lower intensities corresponding to elementary SFB solutions and higher intensities corresponding to higher-order superpositions (or collisions). The properties of the peaks emerging from noise are found to scatter statistically around the analytic properties of the SFB solutions obtained from the NLSE.

Secondly, our analysis of the statistical properties of the peak intensities clearly shows that the Peregrine soliton does not exhibit the statistical rarity to be considered as a rogue wave prototype. Rather, our results confirm that the highest intensity NLSE rogue waves are most appropriately identified as collisions between elementary SFB solutions. This of course raises the question of how the Peregrine soliton should now be interpreted. In this regard, however, we remark that the association of the Peregrine soliton as a rogue wave prototype was initially made on qualitative grounds, simply noting that it is the highest intensity single-breather solution of the NLSE and out of all the elementary solutions, is the only one that is localised both in space and time^11^,^13^. Subsequent studies of the Peregrine soliton as a rogue wave have focused similarly on its intensity characteristics and its growth and decay, and not on its statistical rarity (see e.g. ref. 24). Based on our results, however, it appears that it is inappropriate to consider the Peregrine soliton as a statistical rogue wave, although it will certainly appear as a wave of intensity exceeding the mean in the associated probability distribution.

Finally, it is important to note the importance of developing techniques to allow the real-time measurement of the emergent peaks seen in MI experimentally. The most likely system where this will be possible seems to be optics, since it is possible to observe NLSE dynamics using long optical pulses of picosecond or nanosecond duration where the temporal modulations from MI appear on a timescale at least an order of magnitude faster. The high repetition rate of optical sources (MHz-GHz) also allows the convenient recording of extensive data sets. The use of the dispersive Fourier transform method for real time measurement of spectral fluctuations in optics has already represented a significant experimental advance^28^,^29^,^30^,^31^, and it is possible that the strong temporal-spectral correlations that we have identified here may lead to sensitive real-time measurements of the wings of the MI spectrum that can provide evidence for collision events. However, real-time techniques allowing the full-field characterization of an MI field are still required. In this context, we note that the recently-reported single-shot measurement of the complete temporal intensity and phase of a supercontinuum using an adapted frequency-resolved optical gating technique may represent a very significant step forward^32^.

## Methods

Numerical simulation of noise-seeded MI shown in Fig. 2 is based on a standard split-step scheme. Starting from a noise-perturbed plane wave of normalized amplitude *ψ* (0,*τ*) = 1 + *η* (0,*τ*), the field is propagated up to a distance *ξ*_max_ in a system governed by the pure NLSE. The noise field *η* (*0*,*τ*) here is computed from the inverse Fourier transform of a broadband noise of one-photon per mode spectral density with random phase. This is a common noise source used in the modelling of spectral broadening processes in optics^33^. To have sufficient data for the statistical analysis, the field is propagated up to *ξ*_max_ = 343750 (using 6250000 *ξ*−points), with a temporal window extending to *τ*_max_ = ±69.42 (using 2048 *τ*−points). This yielded a total of 2853669 spontaneously emergent localised structures, and with typically 50 temporal points across each SFB peak. In determining the intensity of these peaks, we used an 8-connected neighborhood regional maximum search. We emphasize that this technique needs to be applied carefully (using interpolation and smoothing/low pass filtering) to avoid the detection of small-scale fluctuations on top of the localised structures as distinct peaks. We further applied a minimum relative threshold of |*ψ*|^2^ = 1 in order to avoid counting the subsidiary maxima of primary peaks.

The temporal width of the evolving field was calculated from the width of the central intensity coherence peak in the intensity autocorrelation function which gives a measure of the average width of the localised peaks in the random MI field. The width *Δ*τ_ac_ is determined as shown in Fig. 5 from the FWHM of the central peak defined between the two adjacent minima. To determine a suitable measure of spectral width, the bandwidth measured in the wings of the spectrum at the −80 dB level was used.

## Author Contributions

S.T., G.G. and M.E. performed the numerical analysis and theory. G.G., F.D. and J.M.D. planned the research project and provided overall supervision. T.G. and C.B. performed independent checking of results. All authors contributed to interpreting the results obtained, and to the writing and review of the final manuscript.

## Additional Information

**How to cite this article**: Toenger, S. *et al*. Emergent rogue wave structures and statistics in spontaneous modulation instability. *Sci. Rep.* **5**, 10380; doi: 10.1038/srep10380 (2015).
